# Role of Evogliptin for Intra‐Class Therapeutic Interchange in Patients With Type 2 Diabetes Mellitus: Subgroup Analysis From A Multi‐Center, Prospective, Observational Study (REVISE Study)

**DOI:** 10.1002/edm2.70270

**Published:** 2026-07-01

**Authors:** Jie‐Eun Lee, Jun Hwa Hong, Sung Hoon Yu, Ki‐Hyun Baek, JaeMyung Yu, Seung Jin Lee, Hyung‐Wook Kim, Kang Seo Park, Jung Han Kim, KyungWan Min, Yong Hwan Lee, Koon Soon Kim, Min Soo Song, Ji Hoon Kim, Jong Chul Won

**Affiliations:** ^1^ Division of Endocrinology and Metabolism, Department of Internal Medicine, CHA Gangnam Medical Center CHA University Seoul Korea; ^2^ Department of Internal Medicine, Daejeon Eulji Medical Center Eulji University Daejeon Korea; ^3^ Department of Internal Medicine, Hanyang University Guri Hospital Hanyang University College of Medicine Guri Korea; ^4^ Department of Internal Medicine, Yeouido St. Mary's Hospital, College of Medicine The Catholic University of Korea Seoul Korea; ^5^ Department of Internal Medicine, Hallym University Kangnam Sacred Heart Hospital Hallym University College of Medicine Seoul Korea; ^6^ Division of Cardiology, Department of Internal Medicine Soonchunhyang University Cheonan Hospital Cheonan Korea; ^7^ Division of Nephrology, Department of Internal Medicine, St. Vincent's Hospital, College of Medicine The Catholic University of Korea Suwon Korea; ^8^ Department of Internal Medicine Sung Ae General Hospital Seoul Korea; ^9^ Division of Endocrinology and Metabolism, Department of Internal Medicine, Nowon Eulji Medical Center Eulji University Seoul Korea; ^10^ Division of Cardiology, Department of Internal Medicine Dong Rae Bong Seng Hospital Busan Korea; ^11^ Department of Internal Medicine Daejeon Endo Internal Medicine Daejeon Republic of Korea; ^12^ Department of Internal Medicine Global Health Care Esoo Hospital Asan Korea; ^13^ Department of Internal Medicine Hong Ik Hospital Seoul Korea; ^14^ Division of Endocrinology and Metabolism, Department of Internal Medicine Gimpo Woori Hospital Gimpo Korea

## Abstract

**Aims:**

While dipeptidyl peptidase‐4 (DPP‐4) inhibitors are commonly considered to have a class effect, emerging evidence suggests intra‐class heterogeneity in pharmacological properties. For patients who fail to achieve target glycemic levels with initial DPP‐4 inhibitors therapy, intra‐class therapeutic interchange may offer an alternative to dose escalation or add‐on therapy. This study evaluated the effectiveness of evogliptin, a novel and potent DPP‐4 inhibitor with a unique binding structure, as a strategy for therapeutic optimization.

**Materials and Methods:**

This follow‐up analysis included patients with HbA1c ≥ 7.0% who had received metformin plus a DPP‐4 inhibitor other than evogliptin for at least 12 weeks and were subsequently switched to evogliptin. Changes in HbA1c and target achievement at 12 and 24 weeks were assessed according to the initial DPP‐4 inhibitors. Multivariable linear regression was performed to identify independent predictors of HbA1c reduction. Additional exploratory subgroup analyses were conducted according to demographic and baseline clinical characteristics.

**Results:**

Among 770 patients in the effectiveness set, mean HbA1c decreased by −0.3% at 12 weeks and −0.5% at 24 weeks (*p* < 0.0001 for both). In the sitagliptin group, reductions were −0.5% and −0.6%, respectively (*p* < 0.0001). At 24 weeks, 25.8% and 50.5% of patients achieved HbA1c targets of < 6.5% and < 7.0%, respectively.

**Conclusions:**

Intraclass therapeutic interchange (ICTI) to evogliptin from other DPP‐4 inhibitors was an effective and well‐tolerated strategy for optimizing glycemic control. These findings suggest that intra‐class therapeutic interchange could be an effective and well‐tolerated strategy for optimizing glycemic control in a clinical setting. However, given the observational nature of this study, further controlled studies are needed to confirm these findings against a comparator group.

**Trial Registration:**

ClinicalTrials.gov NCT04326166

## Graphical abstract

ICTI to evogliptin from other DPP‐4 inhibitors significantly improved glycemic control in patients with type 2 diabetes inadequately controlled on metformin‐based therapy. The treatment was well tolerated, supporting ICTI as a pragmatic optimization strategy in routine clinical practice.

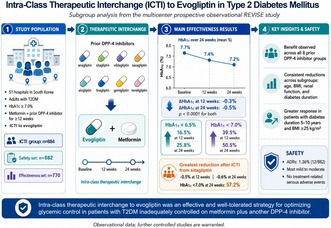

## 1 Introduction

Type 2 diabetes mellitus (T2DM) is a chronic, progressive metabolic disorder characterized by insulin resistance and impaired insulin secretion, and it requires individualized therapy to achieve and maintain glycemic control [1, 2]. Among oral antidiabetic agents, dipeptidyl peptidase‐4 (DPP‐4) inhibitors are widely prescribed because of their efficacy, safety, weight neutrality and low risk of hypoglycemia [3]. These agents improve glycemic control by enhancing incretin activity, stimulating insulin secretion and suppressing glucagon release in a glucose‐dependent manner [4].

Despite the widespread use of DPP‐4 inhibitors, including sitagliptin, saxagliptin, linagliptin, alogliptin, gemigliptin and evogliptin [5], a substantial proportion of patients fail to achieve or maintain adequate glycemic control over time. In clinical practice, therapeutic inertia often arises when glycemic targets are not met, but escalation to other drug classes is limited by cost, tolerability or patient preference [6, 7, 8]. In such scenarios, intraclass therapeutic interchange (ICTI), defined as switching between agents within the same drug class, may represent a pragmatic, low‐risk strategy for treatment optimization.

Although DPP‐4 inhibitors share a common mechanism of action, emerging evidence suggests intra‐class differences in pharmacokinetic and pharmacodynamic properties, including binding affinity, enzyme occupancy and duration of inhibition. These differences raise the possibility that ICTI may result in different clinical responses in certain patients. However, evidence supporting this strategy remains limited, particularly in routine clinical settings. Therefore, evaluating the clinical effectiveness of ICTI represents an important and clinically relevant research question.

Evogliptin is a DPP‐4 inhibitor widely used as monotherapy or in combination with other antidiabetic agents [9]. Although our previous report demonstrated the clinical effectiveness and safety of ICTI to evogliptin in a large‐scale cohort, those findings reflected an aggregate response across a heterogeneous population [10]. Given the pharmacological heterogeneity among DPP‐4 inhibitors, including differences in binding affinity and enzyme occupancy, it is important to determine whether the therapeutic benefit of ICTI is influenced by the specific prior agent used. Therefore, the present study provides a comprehensive analysis of the effectiveness of ICTI to evogliptin according to the eight initial DPP‐4 inhibitors. In addition, we used multivariable regression models to identify independent determinants of treatment response and to refine the clinical rationale for optimizing therapy in patients who remain inadequately controlled on their initial DPP‐4 inhibitor.

We investigated the effectiveness and safety of ICTI to evogliptin from other DPP‐4 inhibitors in patients with T2DM using data from a previously conducted an observational study. We also evaluated potential determinants of treatment response, including demographic characteristics (e.g., age, body mass index (BMI)) and concomitant medications, to identify clinically relevant factors that may help predict treatment outcomes in individual patients. This study may provide evidence supporting the clinical rationale for ICTI among DPP‐4 inhibitors in patients with T2DM.

## 2 Materials and Methods

### 2.1 Study Design

This follow‐up analysis was part of a multicenter, prospective, observational study conducted across 51 hospitals in South Korea (ClinicalTrials.gov NCT04326166). The primary objective of the original study was to evaluate the glucose‐lowering effectiveness and safety of evogliptin in patients with T2DM in a clinical setting. Patients who provided written informed consent and met the eligibility criteria were enrolled in this observational study. Study visits were conducted at baseline and at 12 weeks to evaluate effectiveness and safety, with an additional visit at 24 weeks for further assessment. This analysis specifically focused patients who underwent ICTI to evogliptin, which consisted of patients with haemoglobin (Hb) A1c levels of 7.0% or higher who had previously been taking metformin and a DPP‐4 inhibitor (e.g., anagliptin, sitagliptin, vildagliptin, saxagliptin, alogliptin, linagliptin, gemigliptin or teneligliptin) other than evogliptin for at least 12 weeks and underwent ICTI to evogliptin at baseline because of clinical considerations. When initiating evogliptin, precautions according to the approved labelling–particularly in patients with renal impairment–were taken into account. The study endpoints were changes in HbA1c (%) and the proportions of patients who achieved HbA1c < 6.5% and < 7.0% at 12 and 24 weeks. Additional exploratory analyses were performed according to demographic and baseline clinical characteristics, including age, duration of diabetes, renal function, BMI and use of lipid‐lowering or antihypertensive agents. Safety endpoints included adverse events (AEs), adverse drug reactions (ADRs), serious adverse events (SAEs) and laboratory abnormalities.

The original study was approved by the institutional review boards of all participating centers and conducted in accordance with the Declaration of Helsinki.

### 2.2 Study Population

The inclusion criteria for the ICTI group were adults aged 19 years or older, BMI between 18.5 kg/m [2] and 40 kg/m [2], fasting blood glucose (FBG) < 270 mg/dL and provision of written informed consent. Patients were excluded if they had contraindications to Suganon Tab. or Sugamet XR Tab. or were considered unsuitable by the investigator. The target sample size for the ICTI group was 962, allowing for a 30% dropout rate. The statistical assumptions included a margin of error of 0.065% and a standard deviation of 0.86%, based on data reported by Kim et al. [11].

### 2.3 Data Analysis

We used two datasets for the effectiveness and safety analyses. The effectiveness set included patients who met the inclusion and exclusion criteria, received Suganon Tab. or Sugamet XR Tab. at least once, and had data available for effectiveness analysis. The safety set included patients who received Suganon Tab. or Sugamet XR Tab. at least once and had data available for safety analysis.

The follow‐up analysis was performed within the evogliptin ICTI group according to prior DPP‐4 inhibitor use and baseline characteristics. For effectiveness endpoints, continuous variables were summarized using descriptive statistics, including mean, standard deviation (SD) and median, whereas categorical variables were presented as frequencies and percentages. Unless otherwise specified, all tests were two‐sided, with a significance level of 5%. Changes within groups were analysed using the paired *t*‐test or Wilcoxon's signed rank test when the assumption of normality was not satisfied.

The frequency and percentage of patients who achieved HbA1c < 6.5% and < 7.0% were presented, and 95% confidence intervals (CIs) for the achievement rates were calculated. To identify independent predictors of glycemic response, multivariable linear regression analysis was performed with the change in HbA1c at 24 weeks as the dependent variable. The included covariates were prior DPP‐4 inhibitor type, age, sex, BMI, duration of diabetes, eGFR, baseline HbA1c, use of lipid‐lowering agents and use of antihypertensive agents. Adjusted β coefficients, standard errors and 95% CIs were reported. Safety endpoints were analysed using the frequency, percentage of patients and number of events.

## 3 Results

Of the 884 patients in the ICTI group, 882 were included in the safety set and 770 in the effectiveness set. For the safety analysis, 2 patients were excluded due to non‐administration of evogliptin. For the effectiveness analysis, an additional 112 patients were excluded for the following reasons: lack of effectiveness assessment after baseline (*N* = 63), violation of inclusion/exclusion criteria (*N* = 32) or use of prohibited medication (*N* = 29); 12 patients had overlapping exclusion reasons. The demographic and baseline clinical characteristics are summarized in Table 1. Of these patients, 499 (56.5%) were male and 385 (43.6%) were female. The mean age was 64.7 years and 53.6% were aged ≥ 65 years. The mean duration of T2DM was 9.2 years. The proportion of patients with an estimated glomerular filtration rate (eGFR) < 60 mL/min/1.73 m [2] and 60–< 90 mL/min/1.73 m [2] was 13.6% and 44.7% respectively. The mean BMI was 25.2 kg/m [2], and 51.4% had a BMI ≥ 25 kg/m [2]. In addition, 65.8% of patients were receiving lipid‐lowering agents, and 45.6% were receiving antihypertensive agents at baseline. Regarding concomitant metformin therapy at baseline, the mean daily dose of metformin was generally consistent across the prior DPP‐4 inhibitor subgroups, ranging from 735.1 ± 308.9 mg in the saxagliptin group to 1137.5 ± 410.3 mg in the anagliptin group (Table 1). The median dose of metformin was 1000 mg in most subgroups, except for the saxagliptin (500 mg) and gemigliptin (850 mg) groups.

**TABLE 1:** Demographic and baseline clinical characteristics.

Category	ICTI *N* = 884	Category	ICTI *N* = 884
Sex, *n* (%)		**Use of lipid‐lowering agents, *n*** (**%**)	
*n*	884	*n*	884
Male	499 (56.5)	Yes	582 (65.8)
Female	385 (43.6)	No	302 (34.2)
Age (year)		**Use of antihypertensive agents, *n*** (**%**)	
*n*	884	*n*	884
Mean ± SD	64.7 ± 11.0	Yes	403 (45.6)
Median	66	No	481 (54.4)
Min, Max	21.0, 92.0		
		**Dose of metformin at baseline** (**mg**)	
Age, *n* (%)		**Anagliptin**	Missing value (1)
< 65 years	410 (46.4)	*n*	66
≥ 65 years	474 (53.6)	Mean ± SD	1137.5 ± 410.3
		Median	1000.0
		Min, Max	250.0, 2550.0
Duration of diabetes^a^ (year)		**Sitagliptin**	Missing value (4)
*N*	884	*n*	176
Mean ± SD	9.2 ± 6.8	Mean ± SD	1112.9 ± 424.3
Median	7.8	Median	1000.0
Min, Max	0.0, 43.5	Min, Max	250.0, 2550.0
Duration of diabetes^a^, *n* (%)		**Vildagliptin**	Missing value (0)
< 5 years	271 (30.7)	*n*	65
5 years ≤ ~ < 10 years	276 (31.2)	Mean ± SD	1079.6 ± 511.5
≥ 10 years	337 (38.1)	Median	1000.0
		Min, Max	425.0, 2250.0
eGFR (mL/min/1.73 m^2^)		**Saxagliptin**	Missing value (0)
*n*	631^b^	*n*	84
Mean ± SD	82.3 ± 18.6	Mean ± SD	735.1 ± 308.9
Median	85.8	Median	500.0
Min, Max	24.5, 133.1	Min, Max	250.0, 1500.0
eGFR, *n* (%)		**Alogliptin**	Missing value (9)
< 60 mL/min/1.73 m^2^	86 (13.6)	*n*	30
60 mL/min/1.73 m^2^≤ ~ < 90 mL/min/1.73 m^2^	282 (44.7)	Mean ± SD	1012.5 ± 420.8
		Median	1000.0
≥ 90 mL/min/1.73 m^2^	263 (41.7)	Min, Max	500.0, 1700.0
Height (cm)		**Linagliptin**	Missing value (3)
*n*	884	*n*	147
Mean ± SD	162.7 ± 8.9	Mean ± SD	949.2 ± 468.9
Median	163	Median	1000.0
Min, Max	138.2, 188.0	Min, Max	250.0, 2500.0
Weight (kg)		**Gemilgliptin**	Missing value (1)
*n*	884	*n*	205
Mean ± SD	66.9 ± 10.7	Mean ± SD	854.2 ± 411.2
Median	66	Median	850.0
Min, Max	40.0, 112.0	Min, Max	250.0, 2000.0
BMI (kg/m^2^)		**Teneligliptin**	Missing value (1)
*n*	884	*n*	90
Mean ± SD	25.2 ± 3.1	Mean ± SD	1014.4 ± 410.6
Median	25	Median	1000.0
Min, Max	18.6, 36.4	Min, Max	250.0, 2000.0
BMI, *n* (%)			
< 23 kg/m^2^	211 (23.9)		
23 kg/m^2^ ≤ ~ < 25 kg/m^2^	219 (24.8)		
≥ 25 kg/m^2^	454 (51.4)		

Abbreviations: Max, Maximum; Min, Minimum; SD, Standard deviation.

^a^ Duration of diabetes (years) = ([date of informed consent−date of T2DM] + 1)/365.25.

^b^ Missing eGFR data: 217 patients.

In the effectiveness set (*N* = 770), HbA1c decreased significantly from baseline by −0.3% at 12 weeks and −0.5% at 24 weeks (both *p* < 0.0001). The proportion of patients achieving HbA1c < 6.5% increased from 16.5% (95% CI: 13.9–19.3) at 12 weeks to 25.8% (95% CI: 22.8–29.1) at 24 weeks. Similarly, the proportion achieving HbA1c < 7.0% increased from 39.5% (95% CI: 36.0–43.0) at 12 weeks to 50.5% (95% CI: 46.9–54.1) at 24 weeks. Reductions were observed across all prior DPP‐4 inhibitor groups. The overall results are summarized in Table 2.

**TABLE 2:** Haemoglobin A1c and target achievement rates at baseline, 12 weeks and 24 weeks—Follow‐up analysis based on prior DPP‐4 inhibitor.

Category	Anagliptin	Sitagliptin	Vildagliptin	Saxagliptin	Alogliptin	Linagliptin	Gemigliptin	Teneligliptin	Total
	*N* = 56	*N* = 159	*N* = 56	*N* = 77	*N* = 32	*N* = 125	*N* = 184	*N* = 81	*N* = 770
**HbA1c (%)**									
**Baseline, *n***	56	159	56	77	32	125	184	81	770
Mean ± SD	8.1 ± 1.2	7.7 ± 0.6	7.8 ± 0.6	7.6 ± 0.5	7.8 ± 0.7	7.9 ± 0.8	7.6 ± 0.7	7.8 ± 0.9	7.7 ± 0.8
Median	7.9	7.6	7.6	7.5	7.5	7.6	7.4	7.5	7.5
**Change from baseline to Week 12, *n***	52	144	51	70	29	112	158	77	693
Mean ± SD	−0.2 ± 1.0	−0.5 ± 0.7	−0.4 ± 0.6	−0.3 ± 0.5	−0.3 ± 0.7	−0.2 ± 0.9	−0.3 ± 1.0	−0.2 ± 1.0	−0.3 ± 0.8
Median	−0.2	−0.5	−0.5	−0.4	−0.3	−0.3	−0.3	0	−0.3
*p*‐value*	0.2608**	< 0.0001**	< 0.0001***	< 0.0001***	0.0217***	< 0.0001**	< 0.0001**	0.2876**	< 0.0001**
**Change from baseline to Week 24, *n***	52	144	46	71	28	112	161	66	680
Mean ± SD	−0.1 ± 1.3	−0.6 ± 0.9	−0.6 ± 1.3	−0.5 ± 0.8	−0.4 ± 0.9	−0.4 ± 0.8	−0.5 ± 0.8	−0.6 ± 1.3	−0.5 ± 1.0
Median	−0.2	−0.6	−1	−0.4	−0.5	−0.4	−0.4	−0.4	−0.4
*p*‐value*	0.3491**	< 0.0001***	0.0005**	< 0.0001***	0.0166 ***	< 0.0001***	< 0.0001**	< 0.0001**	< 0.0001**
**Patients Achieving HbA1c < 6.5%**									
**Week 12, *n* (%)**	8 (14.3)	28 (17.6)	7 (12.5)	11 (14.3)	6 (18.8)	23 (18.4)	36 (19.6)	8 (9.9)	127 (16.5)
95% CI	6.4, 26.2	12.0, 24.4	5.2, 24.1	7.4, 24.1	7.2, 36.4	12.0, 26.3	14.1, 26.0	4.4, 18.5	13.9, 19.3
**Week 24, *n* (%)**	6 (10.7)	45 (28.3)	19 (33.9)	23 (29.9)	9 (28.1)	29 (23.2)	46 (25.0)	22 (27.2)	199 (25.8)
95% CI	4.0, 21.9	21.5, 36.0	21.8, 47.8	20.0, 41.4	13.7, 46.7	16.1, 31.6	18.9, 31.9	17.9, 38.2	22.8, 29.1
**Patients Achieving HbA1c < 7.0%**									
**Week 12, *n* (%)**	17 (30.4)	69 (43.4)	17 (30.4)	37 (48.1)	15 (46.9)	50 (40.0)	84 (45.7)	15 (18.5)	304 (39.5)
95% CI	18.8, 44.1	35.6, 51.5	18.8, 44.1	36.5, 59.7	29.1, 65.3	31.3, 49.1	38.3, 53.1	10.8, 28.7	36.0, 43.0
**Week 24, *n* (%)**	16 (28.6)	91 (57.2)	32 (57.1)	45 (58.4)	17 (53.1)	54 (43.2)	99 (53.8)	35 (43.2)	389 (50.5)
95% CI	17.3, 42.2	49.2, 65.0	43.2, 70.3	46.6, 69.6	34.7, 70.9	34.4, 52.4	46.3, 61.2	32.2, 54.7	46.9, 54.1

*Note:* *Within‐group comparison (baseline to 12 and 24 weeks): paired *t*‐test (**), Wilcoxon signed rank test (***).

Abbreviations: CI, confidence interval; SD, standard deviation.

The number of patients in each prior DPP‐4 inhibitor group was as follows: anagliptin (*N* = 56), sitagliptin (*N* = 159), vildagliptin (*N* = 56), saxagliptin (*N* = 77), alogliptin (*N* = 32), linagliptin (*N* = 125), gemigliptin (*N* = 184) and teneligliptin (*N* = 81). Glycemic outcomes at 12 and 24 weeks according to the previously used DPP‐4 inhibitor are detailed in Table 2. Patients previously treated with sitagliptin showed the greatest reduction in HbA1c from baseline, with mean ± SD [median] changes of −0.5 ± 0.7 [−0.5] at 12 weeks (*p* < 0.0001) and −0.6 ± 0.9 [−0.6] at 24 weeks (*p* < 0.0001), and they also had the highest proportion of patients achieving HbA1c < 7.0% at 12 weeks (43.4%) and 24 weeks (57.2%). Patients previously treated with saxagliptin or vildagliptin also showed moderate decreases in HbA1c levels from baseline at both 12 and 24 weeks. Overall, HbA1c levels decreased at 12 and 24 weeks following ICTI to evogliptin from any of the prior DPP‐4 inhibitors evaluated in this study.

**FIGURE 1:**
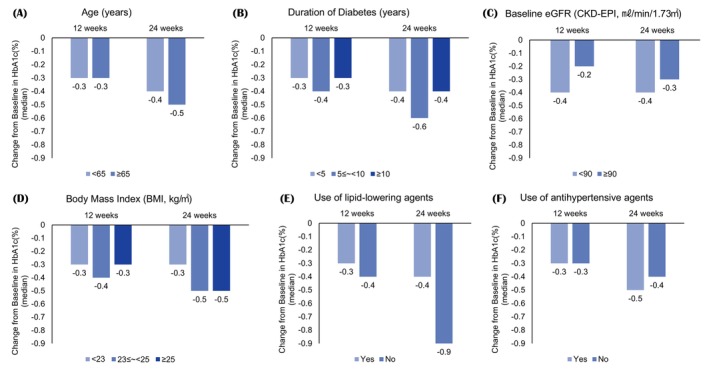
Changes in glycated haemoglobin (HbA1c, %) from baseline at 12 and 24 weeks: Follow‐up analysis according to demographic and baseline clinical characteristics.

Further analysis provided additional insight into glycemic outcomes according to specific baseline characteristics. Patients aged < 65 years showed reductions of −0.3% at 12 weeks and −0.4% at 24 weeks, whereas those aged ≥ 65 years showed reductions of −0.3% and −0.5%, respectively (Figure 1A). HbA1c reductions from baseline were statistically significant at both 12 and 24 weeks in each age group (*p* < 0.0001).

Patients with a diabetes duration of 5–10 years showed the greatest reductions in HbA1c from baseline, with decreases of −0.4% at 12 weeks and −0.6% at 24 weeks. Patients with a duration of < 5 years showed reductions of −0.3% at 12 weeks and −0.4% at 24 weeks, whereas those with a duration of ≥ 10 years showed similar reductions of −0.3% and −0.4%, respectively (Figure 1B). HbA1c reductions from baseline were statistically significant at both 12 and 24 weeks across all three duration groups (*p* < 0.0001).

HbA1c reductions from baseline were also evaluated according to renal function, as indicated by eGFR. Patients with eGFR < 90 mL/min/1.73 m [2] showed reductions of −0.4% at both 12 and 24 weeks, whereas those with eGFR ≥ 90 mL/min/1.73 m [2] showed smaller reductions of −0.2% and −0.3% respectively (Figure 1C). These reductions were statistically significant in each renal function group at both 12 and 24 weeks (*p* < 0.0001).

Glycemic outcomes also differed according to BMI. Patients with BMI < 23 kg/m [2] showed HbA1c reductions from baseline of −0.3% at both 12 and 24 weeks, whereas those with BMI ≥ 25 kg/m [2] showed reductions of −0.3% and −0.5%, respectively (Figure 1D). HbA1c reductions from baseline were statistically significant at both 12 and 24 weeks in each BMI group (*p* < 0.0001).

Additionally, glycemic outcomes differed according to the use of concomitant medications, including lipid‐lowering and antihypertensive agents. Patients receiving lipid‐lowering agents showed smaller reductions (−0.3% at 12 weeks and −0.4% at 24 weeks) than those not receiving lipid‐lowering agents (−0.4% and −0.9%, respectively) (Figure 1E). Patients receiving antihypertensive agents showed reductions of −0.3% at 12 weeks and −0.5% at 24 weeks, whereas those not receiving antihypertensive agents showed changes of −0.3% and −0.4%, respectively (Figure 1F). These reductions were statistically significant at both time points among patients receiving lipid‐lowering or antihypertensive agents (*p* < 0.0001).

To further evaluate determinants of glycemic response, multivariable linear regression analysis was conducted (Table 3). Baseline HbA1c was the strongest independent predictor of HbA1c reduction (*β* = −0.4895, *p* < 0.0001), indicating that patients with higher initial HbA1c experienced greater improvement after ICTI to evogliptin. Longer duration of diabetes was independently associated with a smaller HbA1c reduction (*β* = 0.0138, *p* = 0.0203). Regarding prior DPP‐4 inhibitor exposure, ICTI from anagliptin was associated with a significantly smaller HbA1c reduction than ICTI from sitagliptin (*β* = 0.5320, *p* = 0.0015). No significant differences were observed for the other DPP‐4 inhibitors. Age, sex, BMI, renal function and concomitant medication use were not significantly associated with HbA1c change.

**TABLE 3:** Multivariable linear regression analysis of change in HbA1c from baseline to 24 weeks.

	Parameter estimate	Standard error	*t*	*p*	Lower 95% CI	Upper 95% CI
Intercept	2.5009	0.7059	3.54	0.0004	1.1139	3.8879
Prior DPP‐4 inhibitor type						
Sitagliptin	Ref					
Anagliptin	0.5320	0.1666	3.19	0.0015	0.2046	0.8594
Vildagliptin	0.1674	0.1888	0.89	0.3757	−0.2036	0.5385
Saxagliptin	0.1883	0.1507	1.25	0.2119	−0.1077	0.4844
Alogliptin	0.1703	0.2013	0.85	0.3980	−0.2252	0.5657
Linagliptin	0.2526	0.1330	1.90	0.0581	−0.0087	0.5138
Gemigliptin	0.1255	0.1132	1.11	0.2682	−0.0970	0.3480
Teneligliptin	0.0982	0.1529	0.64	0.5210	−0.2023	0.3987
Sex						
Male	0.0943	0.0806	1.17	0.2424	−0.0640	0.2526
Female	Ref					
Age						
years	−0.0020	0.0045	−0.43	0.6663	−0.0109	0.0070
BMI						
kg/m^2^	0.0039	0.0134	0.30	0.7679	−0.0223	0.0302
Duration of diabetes^a^						
years	0.0138	0.0059	2.33	0.0203	0.0022	0.0255
eGFR						
mL/min/1.73 m^2^	0.0046	0.0025	1.83	0.0683	−0.0003	0.0096
Baseline HbA1c						
%	−0.4895	0.0498	−9.83	< 0.0001	−0.5874	−0.3917
Use of lipid‐lowering agents						
Yes	0.1486	0.0973	1.53	0.1272	−0.0425	0.3397
No						
Use of antihypertensive agents						
Yes	0.0233	0.0822	0.28	0.7772	−0.1382	0.1847
No	Ref					

Abbreviation: CI, confidence interval.

^a^ Duration of diabetes (years) = (date of informed consent—date of type 2 diabetes diagnosis +1)/365.25.

With regard to safety outcomes, ADRs were reported in a small proportion of patients, and most were classified as mild to moderate in severity. SAEs were rare, and no serious adverse events related to evogliptin treatment were identified. A detailed summary of safety outcomes is presented in Table 4.

**TABLE 4:** Adverse drug reactions and serious adverse events reported over 24 weeks.

Category	ICTI *N* = 882	Category	ICTI *N* = 882
**Adverse drug reaction, *n* (%) (event)**	**12 (1.36) (13)**	**Serious adverse event, *n* (%) (event)**	**12 (1.36) (14)**
**System organ class/preferred term** ^**a**^, ***n* (%) (event)**		**System organ class/preferred term** ^**a**^, ***n* (%) (event)**	
**Gastrointestinal disorders**	**5 (0.57) (5)**	**Metabolism and nutrition disorders**	**1 (0.11) (1)**
Abdominal discomfort	1 (0.11) (1)	Hyperglycaemia	1 (0.11) (1)
Diarrhoea	1 (0.11) (1)		
Nausea	1 (0.11) (1)	**Neoplasms benign, malignant and unspecified (incl cysts and polyps)**	**1 (0.11) (1)**
Dry mouth	1 (0.11) (1)		
Gastrooesophageal reflux disease	1 (0.11) (1)	Gastric cancer	1 (0.11) (1)
**Skin and subcutaneous tissue disorders**	**1 (0.11) (1)**	**Cardiac disorders**	**2 (0.23) (2)**
Urticaria	1 (0.11) (1)	Cardiac failure	1 (0.11) (1)
		Angina unstable	1 (0.11) (1)
**Metabolism and nutrition disorders**	**2 (0.23) (2)**	**Injury, poisoning and procedural complications**	**2 (0.23) (2)**
Hypoglycaemia	1 (0.11) (1)	Femoral neck fracture	1 (0.11) (1)
Decreased appetite	1 (0.11) (1)	Meniscus cyst	1 (0.11) (1)
**Investigations**	**2 (0.23) (2)**	**Nervous system disorders**	**2 (0.23) (3)**
Blood creatinine increased	1 (0.11) (1)	Headache	1 (0.11) (1)
Blood glucose increased	1 (0.11) (1)	Intracranial aneurysm	1 (0.11) (1)
		Thalamic infarction	1 (0.11) (1)
**Nervous system disorders**	**1 (0.11) (1)**	**Hepatobiliary disorders**	**2 (0.23) (2)**
Headache	1 (0.11) (1)	Hepatic mass	1 (0.11) (1)
		Liver injury	1 (0.11) (1)
**General disorders and administration site conditions**	**1 (0.11) (1)**	**Infections and infestations**	**1 (0.11) (1)**
Gait disturbance	1 (0.11) (1)	Sinusitis	1 (0.11) (1)
**Psychiatric disorders**	**1 (0.11) (1)**	**Renal and urinary disorders**	**1 (0.11) (1)**
Insomnia	1 (0.11) (1)	Ureterolithiasis	1 (0.11) (1)
		**Respiratory, thoracic and mediastinal disorders**	**1 (0.11) (1)**
		Pleural effusion	1 (0.11) (1)

*Note:* Multiple events per patient were possible.

^a^ MedDRA (27.0).

## 4 Discussion

This study investigated the effectiveness and safety of ICTI to evogliptin from other dipeptidyl peptidase‐4 (DPP‐4) inhibitors in patients with T2DM who had inadequate glycemic control (HbA1c ≥ 7.0%) despite concurrent metformin therapy. Although DPP‐4 inhibitors are widely used because of their efficacy and safety profile, clinical evidence regarding the effects of ICTI within the DPP‐4 inhibitor class remains limited, especially in routine clinical settings.

The key finding was the clinically meaningful reduction in HbA1c observed after ICTI to evogliptin; the mean change in HbA1c was −0.5 ± 1.0 at 24 weeks in the overall patient cohort (*p* < 0.0001), consistent with the established efficacy and safety profile of evogliptin [12]. Furthermore, a meaningful proportion of patients achieved target glycemic control, with 50.5% achieving HbA1c < 7.0% at 24 weeks.

While these results provide real‐world evidence of HbA1c reduction after ICTI to evogliptin, they should be interpreted with caution as the lack of a control group (e.g., patients continuing their prior therapy) precludes a definitive conclusion that the improvement was solely due to the pharmacological properties of evogliptin. Although all gliptins target the DPP‐4 enzyme, they have distinct chemical structures, which lead to differences in binding kinetics and binding sites [13]. For example, evogliptin interacts with the S2‐extensive subsite of the DPP‐4 enzyme in a manner that differs from that of older DPP‐4 inhibitors [14]. We hypothesize that, in patients whose glycemic control was inadequate on a prior DPP‐4 inhibitor, ICTI to evogliptin may achieve more complete or sustained enzyme occupancy. This differential inhibition profile, whether by reaching a higher inhibition threshold or maintaining a longer duration of effective inhibition, could lead to a greater and more sustained increase in active GLP‐1, thereby contributing to the additional clinically significant HbA1c reduction observed after ICTI.

The observed effectiveness of evogliptin in patients undergoing ICTI to evogliptin may be attributed to its distinct pharmacological profile compared to other DPP‐4 inhibitors. Unlike earlier DPP‐4 inhibitors, evogliptin interacts with the S2‐extensive subsite of the DPP‐4 enzyme, a feature that may contribute to its high binding affinity, and evogliptin demonstrates a significantly lower IC50 of approximately 0.9 nM, representing nearly 20‐fold greater inhibitory activity than sitagliptin (~18 nM). In addition, while agents like sitagliptin have a half‐life of roughly 12.4 h, evogliptin exhibits an extended half‐life of 33–39 h, thus this profile allows evogliptin to maintain a DPP‐4 inhibition rate of over 80% even 24 h post‐dose, potentially mitigating the ‘trough‐period’ glycemic escape that can occur with shorter‐acting inhibitors. While these pharmacological characteristics provide a biologically plausible rationale for the clinical benefits observed after therapeutic interchange, it is important to note that direct pharmacodynamic markers, such as enzyme occupancy or active GLP‐1 levels, were not measured in this study. Thus, these mechanistic links remain speculative and warrant further investigation in controlled experimental settings [15].

The observed HbA1c reduction was consistent across all eight prior DPP‐4 inhibitor groups. Notably, the sitagliptin subgroup showed the most pronounced reduction (−0.6 ± 0.9 at 24 weeks; *p* < 0.0001) and the highest achievement rate for HbA1c < 7.0% (57.2%), suggesting the potential clinical utility of evogliptin as an alternative for patients with suboptimal responses to prior DPP‐4 inhibitors. In contrast, prior use of anagliptin was associated with a smaller HbA1c reduction than sitagliptin, whereas the other DPP‐4 inhibitors did not show significant differences. Although this finding may suggest heterogeneity in treatment response, it should be interpreted cautiously because of possible residual confounding and baseline differences among groups, and the limited sample size of some subgroups. Furthermore, our subgroup analysis identified potential determinants of treatment response, with greater reductions observed in patients with a diabetes duration of 5–10 years or a BMI ≥ 25 kg/m [2].

The finding in patients with BMI ≥ 25 kg/m [2] is particularly noteworthy, as DPP‐4 acts as an adipokine whose expression and secretion are significantly upregulated in the visceral adipose tissue of individuals with obesity [16, 17]. Given that obesity is associated with higher baseline circulating DPP‐4 levels, the potent and sustained inhibitory action of evogliptin may be especially effective in neutralizing the increased enzymatic burden in this population.

Regarding diabetes duration, the significantly greater HbA1c reduction observed in patients with a 5–10‐year history of T2DM suggests a therapeutic window in which ß‐cell function remains sufficiently preserved to respond to enhanced incretin stimulation. It is well established that ß‐cell function progressively declines by approximately 4%–5% annually from the time of diagnosis [18]. Our findings are consistent with a meta‐analysis indicating that a shorter duration of diabetes is a significant predictor of greater HbA1c lowering with DPP‐4 inhibitors [19]. While patients with a duration exceeding 10 years may have more advanced ß‐cell exhaustion, the 5–10‐year group may represent a population in which the sustained and robust inhibitory action of evogliptin can effectively leverage the remaining insulinotropic potential of the incretin system. This suggests that ICTI to a more potent DPP‐4 inhibitor, such as evogliptin, may be particularly effective before the critical threshold of total ß‐cell failure is reached.

Regarding safety, the incidence of ADRs reported in the safety set (*N* = 882) was low, at 1.36% (12 patients). No SAEs related to evogliptin treatment were identified, further supporting its favourable tolerability profile.

A major strength of this study lies in its observational design, which provides pragmatic evidence across a broad range of patients ICTI from multiple DPP‐4 inhibitors. However, several limitations must be acknowledged. First and foremost, the absence of a control group who maintained their previous DPP‐4 inhibitor therapy means we cannot exclude the possibility of a “Hawthorne effect” or the impact of intensified lifestyle modifications following a medication change. Furthermore, as a non‐randomized observational study, potential selection bias by investigators and the “order effect” of ICTI at a specific time point could have influenced the outcomes.

In conclusion, ICTI to evogliptin from other DPP‐4 inhibitors was associated with significant and sustained HbA1c reduction and a high rate of target achievement in patients with type 2 diabetes inadequately controlled on metformin‐based therapy. While these observational findings suggest that intra‐class differences might play a role in glycemic response, they primarily support ICTI to evogliptin as a pragmatic, low‐risk strategy for treatment optimization in routine clinical practice, particularly when other intensification options are limited. Given its favourable tolerability and ease of implementation, ICTI to evogliptin may represent a pragmatic, low‐risk therapeutic optimization strategy, particularly for patients in whom treatment intensification with other drug classes is not feasible. Future controlled studies are warranted to confirm these findings and to better define the patient subgroups most likely to benefit from this approach.

## Author Contributions

**JaeMyung Yu:** investigation, resources. **Jun Hwa Hong:** writing – original draft, data curation, formal analysis, writing – review and editing, validation, investigation, methodology. **Ki‐Hyun Baek:** investigation, resources. **Sung Hoon Yu:** resources, investigation. **Jong Chul Won:** conceptualization, investigation, methodology, supervision, project administration, writing – review and editing, funding acquisition. **Jung Han Kim:** investigation, resources. **KyungWan Min:** investigation, resources. **Yong Hwan Lee:** investigation, resources. **Seung Jin Lee:** investigation, resources. **Hyung‐Wook Kim:** investigation, resources. **Kang Seo Park:** investigation, resources. **Ji Hoon Kim:** resources, investigation. **Min Soo Song:** resources, investigation. **Koon Soon Kim:** investigation, resources. **Jie‐Eun Lee:** writing – original draft, writing – review and editing, formal analysis, data curation, investigation, validation, visualization.

## Funding

This work was supported by Dong‐A ST, Co., Ltd. Seoul, Republic of Korea.

## Conflicts of Interest

The authors declare no conflicts of interest.

## Data Availability

The data that support the findings of this study are available from the corresponding author upon reasonable request.
